# Autologous bone marrow-derived mononuclear cells transplantation in type 2 diabetes mellitus: effect on β-cell function and insulin sensitivity

**DOI:** 10.1186/s13098-017-0248-7

**Published:** 2017-07-04

**Authors:** Shobhit Bhansali, Pinaki Dutta, Mukesh Kumar Yadav, Ashish Jain, Sunder Mudaliar, Meredith Hawkins, Anura V. Kurpad, Deepak Pahwa, Ashok Kumar Yadav, Ratti Ram Sharma, Vivekanand Jha, Neelam Marwaha, Shipra Bhansali, Anil Bhansali

**Affiliations:** 1 Department of Endocrinology, Post Graduate Institute of Medical Education and Research, Sector-12, Chandigarh, 160012 India; 2 Department of Radiodiagnosis, Post Graduate Institute of Medical Education and Research, Chandigarh, India; 3 Department of Transfusion Medicine, Post Graduate Institute of Medical Education and Research, Chandigarh, India; 4 Department of Medicine, University of California, San Diego, La Jolla, CA USA; 5 Diabetes Research and Training Center and Division of Endocrinology, Department of Medicine, Albert Einstein College of Medicine, Bronx, NY USA; 6 Department of Physiology, St. John’s Medical College, Bangalore, India; 7 Department of Nephrology/Translational and Regenerative Medicine, Post Graduate Institute of Medical Education and Research, Chandigarh, India; 8 Department of Experimental Medicine and Biotechnology, Post Graduate Institute of Medical Education and Research, Chandigarh, India

**Keywords:** T2DM, Stem cells, Autologous bone marrow-derived mononuclear cells

## Abstract

**Background:**

Insulin resistance and insulin deficiency are the cardinal defects in the pathogenesis of type 2 diabetes mellitus (T2DM). Despite the plethora of anti-diabetic medications, drugs specifically targeting the β-cells are still desired. Stem cell therapy has emerged as a novel therapeutics strategy to target β-cells; however, their mechanism of action has not been well defined. This study aims to examine the efficacy and safety of autologous bone marrow-derived mononuclear cells (ABM-MNCs) transplantation in T2DM, and explores the mechanistic insights into stem cells action through metabolic studies.

**Methods:**

Seven T2DM patients with the duration of disease ≥5 years, receiving triple oral anti-diabetic drugs along with insulin (≥0.4 IU per kg per day) and HbA1c ≤ 7.5% (≤58.0 mmol/mol) were enrolled for ABM-MNCs administration through a targeted approach. The primary end-point was a reduction in insulin requirement by ≥50% from baseline, while maintaining HbA1c < 7.0% (<53.0 mmol/mol) with improvement in insulin secretion, and/or insulin sensitivity after ABM-MNCs transplantation.

**Results:**

Six out of 7 (90%) patients achieved the primary end-point. At 6 months, there was a significant reduction in insulin requirement by 51% as compared to baseline (p < 0.003). This was accompanied by a significant increase in the 2nd phase C-peptide response during hyperglycemic clamp (p = 0.018), whereas there were no significant alterations in insulin sensitivity and glucose disposal rate during hyperinsulinemic–euglycemic clamp relative to the baseline. Other measures of β-cell indices like HOMA-β, and stimulated C-peptide response to glucagon and mixed meal tolerance test were non-contributory.

**Conclusion:**

ABM-MNCs transplantation results in significant reduction in insulin doses and improvement in C-peptide response in patients with T2DM. Metabolic studies may be more useful than conventional indices to predict β-cell function in patients with advanced duration of T2DM.

*Trial registration* **-**Clinicaltrials.gov NCT01759823

**Electronic supplementary material:**

The online version of this article (doi:10.1186/s13098-017-0248-7) contains supplementary material, which is available to authorized users.

## Background

Progressive understanding of the pathogenesis of type 2 diabetes mellitus (T2DM) from “dual defect” to “ominous octet” have resulted in the development of the plethora of anti-diabetic medications in the management of T2DM. The recommended first-line therapy for the management of T2DM is life-style modification and metformin. However, metformin loses its efficacy with advancing duration of diabetes, suggesting the decline in β-cell function is progressive and inevitable. Eventually, many patients require a combination of multiple drugs and/or insulin therapy targeting different pathogenetic mechanisms. Although, these therapies help to maintain glycemic control but do not reverse the pathophysiology of the disease, as drugs specifically targeting β-cells are still lacking [1]. This has prompted the clinical investigators to explore the newer therapeutic strategies like cell-based therapy, such as bone marrow-derived adult stem cells, which comprises of hematopoietic stem cells (HSCs) and mesenchymal stem cells (MSCs).

Bone marrow-derived adult stem cells possess homing-in, differentiation, and transdifferentiation properties, which may be useful in improving the β-cell function/mass. Several studies on hematopoietic and mesenchymal stem cells transplantation in animal model demonstrated a significant reduction in blood glucose level through mechanisms such as improved β-cell function, islet neogenesis, and possibly enhancement in insulin sensitivity as well [2–4]. In human, several studies have revealed that use of mononuclear cells (MNCs) entailing hematopoietic stem cells (HSCs), and MSCs transplantation led to a significant decrease in insulin doses and fasting plasma glucose; however, mechanisms involved in the improvement of β-cell function/mass are lacking [5–9]. Various investigators have used different modalities to assess the β-cell function [HOMA-β, fasting C-peptide, stimulated C-peptide response to mixed meal tolerance test (MMTT) and glucagon] and insulin resistance (HOMA-IR, insulin sensitivity (HOMA-S) and Matsuda index); however, the results obtained from these various indices were varying [5, 8, 9]. Nevertheless, the information on the mechanism of action elucidated through metabolic studies following stem cell therapy in human is not available.

Recently, we have published 1-year follow-up data of patients with T2DM following autologous bone marrow-derived mesenchymal stem cells (ABM-MSCs) and autologous bone marrow-derived mononuclear cells (ABM-MNCs) transplantation. It showed an improvement in insulin sensitivity and augmented C-peptide response following ABM-MSCs and ABM-MNCs transplantation, respectively by the hyperglycemic clamp [10]. However, hyperglycemic clamp is considered as a “gold standard” method to assess the β-cell function, but not for the measurement of insulin sensitivity. Therefore, we used the “gold standard” methods to assess the β-cell function and insulin sensitivity by hyperglycemic and hyperinsulinemic–euglycemic clamp, respectively, in addition to conventional measures in seven patients who received ABM-MNCs transplantation.

We hypothesized that autologous bone marrow-derived mononuclear cells (ABM-MNCs) in patients with T2DM would lead to decrease in insulin requirement with sustained euglycemia and this may be modulated through either improvement in β-cell function or insulin sensitivity or both. Therefore, this study plans to elucidate the mechanistic insights into stem cells actions through the different dynamic test for β-cell reserve and insulin sensitivity including metabolic studies.

## Methods

### Study design

The patients were screened at endocrine out-patient department of the Postgraduate Institute of Medical Education and Research, Chandigarh, India. Informed consent was obtained from the study subjects, Stem Cell Ethics Committee of the institute approved the study and trial was registered at Clinicaltrials.gov (ID number, NCT01759823). The inclusion criteria were patients of either sex with T2DM, age between 30 and 65 years with duration of diabetes ≥5 years, and failure to achieve HbA1c ≤ 7.5% (≤58.0 mmol/mol), while receiving triple oral anti-diabetic drugs in optimal doses along with insulin (≥0.4 IU per kg per day) for the last 6 months. Prior to enrollment, patients were on stable doses of vildagliptin, metformin, pioglitazone and insulin (≥0.4 IU per kg per day) during run-in-period for at least 3 months with HbA1c ≤ 7.5% (≤58.0 mmol/mol). Patients with T1DM, glutamate decarboxylase-65 seropositive, active infections, acute coronary syndrome, abnormal liver, renal function tests in the past 3 months, and malignancy were excluded. Seven patients were subjected to C-peptide response to glucagon and mixed meal tolerance test. These patients further underwent hyperinsulinemic–euglycemic clamp and hyperglycemic clamp to assess in vivo insulin secretion and sensitivity, respectively, before and after ABM-MNCs transplantation.

### Baseline evaluation

All subjects underwent clinical and biochemical assessment regarding glycemic control and for micro-, and macrovascular complications.

Each subject underwent a fasting C-peptide, glucagon stimulation test, mixed meal tolerance test, HOMA-β, HOMA-IR, HOMA-S, hyperinsulinemic–euglycemic clamp study to assess in vivo insulin sensitivity and hyperglycemic clamp to assess endogenous insulin secretion, in random order within a 1- to 2-week interval. Each test was performed after a 10–12 h overnight fast.

Subjects were requested to refrain from vigorous exercise, and anti-diabetic medications were omitted 24 h prior and reported at 630 h after an overnight fast of 10 h for both the clamp studies. The plasma glucose was determined by the glucose oxidase method on glucose analyzer (GM9D, Analox instruments, London, UK). C-peptide and plasma insulin level were estimated by electrochemiluminescence immunoassay (Elecsys Roche, Mannheim, Germany).

### Efficacy studies

#### Estimation of homeostatic model assessment

The updated version of the homeostatic model assessment (HOMA) of insulin resistance (HOMA-IR), β cell function (HOMA-β) and insulin sensitivity (HOMA-S) was assessed as per the standard formulae [11, 12].

#### Glucagon stimulated C-peptide

The test was performed in fasting state after intravenous (IV) administration of 1 mg glucagon, and blood samples were drawn at −15 and 0, and 6 min after injection.

#### Mixed meal tolerance test

The mixed meal (10 kcal/kg, Ensure, Abbott Nutrition, Abbott Laboratories, India) dissolved in 500 mL of water was administered to the patient within 10 min. The blood samples were collected at 0, 30, 60, 90 and 120 min and plasma glucose and C-peptide concentrations were measured, respectively. Peak C-peptide response was measured as the highest observed C-peptide level between 0 and 120 min, and area under the curve was calculated following the trapezium rule.

#### Hyperinsulinemic–euglycemic clamp

Subjects were admitted to the hospital before the study for low-dose overnight insulinization. All subjects at inclusion for the clamp studies had plasma glucose <11.1 mmol/L. The insulin infusion rate was adjusted according to an algorithm based on hourly blood glucose measurement [13].

Insulin was infused intravenously based on surface area/min at a constant rate of 40 mU/m^2^/min to raise the plasma insulin concentration to about 100 μU/mL. The glucose infusion rate was adjusted to maintain at steady state of 4.9 mmol/L. Blood samples for glucose and insulin measurements were collected every 5 and 20 min, respectively [14].

#### Hyperglycemic clamp

Glucose solution (20%) was rapidly infused intravenously to raise the plasma glucose level to the target level (15.5 mmol/L) for the next 180 min. Blood samples for glucose and C-peptide were drawn at −5, 0, 2, 4, 6, 8, 10, 30, 60, 120, 140, 160, and 180 min in relation to the initiation of glucose infusion. The 1st phase C-peptide (nmol/L) response was analyzed as the area under the curve from 2 to 10 min, and the 2nd phase C-peptide response as the AUC from 120 to 180 min during the hyperglycemic clamp. The insulin sensitivity index (ISI) was calculated by dividing the steady state (140–180 min) for mean glucose infusion rate (μmoles per kg body weight per min) by the mean insulin concentration (pmol/L) [14, 15].

### Preparation of ABM-MNCs

Approximately 200–250 mL of bone marrow was harvested from the posterior superior iliac spine under local anesthesia following sterile conditions. The mononuclear cells (MNCs) were isolated by centrifugation after layering on density-gradient medium (Ficoll-Hypaque, Sigma-Aldrich, St. Louis, MO, USA). These cells were washed using phosphate-buffered saline (PBS; Himedia Laboratories Private Limited, Mumbai, India) and resuspended in normal saline with a final volume of 8–10 mL. Further, 1 mL of aliquots were taken for MNCs count, CD34^+^ cell count and cell viability. MNCs count was done by using Automated Hematology Analyzer (Orion-60, Ocean Medical Technology, India). CD34^+^ cell count was done on flow cytometer using a fluorochrome labeled monoclonal antibodies like CD45-FITC and anti-CD34-PE. Viability testing was done by a trypan blue (Sigma-Aldrich) exclusion test using haemocytometer (Sigma-Aldrich). Five milliliters of final wash supernatant was used for sterility testing (aerobic and anaerobic culture, Bactec, BD Bioscience) [5, 6, 10].

### Stem cells transplantation

The procedure of stem cell transplantation has been described previously [5, 6, 10]. Briefly, a 5 F catheter (Sim1, Beacon^®^, USA) was selectively navigated through transfemoral route into the celiac trunk. Within the Sim1 catheter, another caliber catheter (Progreat microcatheter, Terumo, Japan) was selectively advanced into superior pancreatico-duodenal (SPD) artery and cells were injected accordingly, unless vascular anomalies were detected. Post-procedure, the patients were monitored for any immediate complications for the next 24 h.

### Follow-up

All patients were followed up every 2 weekly for the first month, then monthly up to 6 months. Lifestyle modification advice was reinforced during each visit to all the patients. Self-monitoring of blood glucose (SMBG) was advised at least five points per week and at the time of suspected hypoglycemia. Fasting plasma glucose (FPG) levels were targeted between 5.0 and 7.2 mmol/L, postmeal glucose levels <9.9 mmol/L and HbA1c < 7.0% (<53.0 mmol/mol). Insulin doses were tapered whenever the patient reported hypoglycemic episodes or when the FPG was <4.4 mmol/L and a postmeal glucose <10.0 mmol/L, without any alterations in oral anti-diabetic drugs. Hyperinsulinemic–euglycemic clamp, hyperglycemic clamp, and mixed meal tolerance test were repeated after 6 months, and glucagon-stimulated C-peptide test was done at 3 and 6 months. All patients were followed for a period of 6 months.

### Outcomes

The primary end point was a reduction in insulin requirement by ≥50%, while maintaining HbA1c < 7.0% (<53.0 mmol/mol) [5, 10]. The secondary end points included alterations in weight, HbA1c, metabolic indices including stimulated C-peptide and insulin sensitivity as compared to the baseline.

### Statistical analysis

All the data are expressed as median and interquartile range. Baseline and post-treatment data within the groups were compared using Friedman’s test with post hoc Wilcoxon’s signed rank test (p value corrected using Bonferroni procedure). The data within the groups were analyzed using the Wilcoxon signed rank test for continuous variables (for two visits). The p-value <0.05 was considered significant. The statistical analysis was carried out using the SPSS version 22 for window (SPSS Inc., Chicago, USA).

## Results

The median age of the patients (n = 7, 6 male) was 46.0 (41.0–54.0) years with duration of diabetes 15.0 (12.0–15.0) years. All patients had neuropathy, five had microalbuminuria and six had nonproliferative diabetic retinopathy (NPDR). Five patients had hypertension. The median duration of insulin treatment and insulin dose was 3.0 (3.0–5.0) years and 76.0 (50.0–92.0) IU/day, respectively. The baseline HbA1c of the group was 6.8% (6.4–7.5%). All patients completed the follow-up for 6 months. The median volume of the bone marrow harvested for transplantation was 224.0 (213.0–227.0) mL, which yielded 1.2 (1.0–1.4) × 10^9^ MNCs (Additional file 1: Table S1) and 2.0 (0.8–2.3) × 10^7^ of these MNCs expressed CD34^+^. In all patients, MNCs infusion was carried out in SPD artery (Additional file 1: Table S1).

After SCT, 6 out of 7 (90%) patients fulfilled the primary end point with a decrease in the total daily insulin requirement by 51% at 6 months (p = 0.003), while maintaining HbA1c < 7% (<53 mmol/mol) till the end of the study period (Table 1).

**Table 1:** Comparison of clinical and biochemical parameters following ABM-MNCs treatment

Parameters	Baseline (n = 7)	3 months (n = 7)	6 months (n = 7)	p value
Primary end point achieved	–	4	6	–
Weight (kg)	84.5(67.7–87.3)	82.7(69.6–88.5)	82.3 (70.1–85.3)	0.964
BMI (kg/m^2^)	29.3 (24.6–30.8)	28.6 (24.9–30.8)	28.6 (25.3–30.9)	0.964
Insulin requirement (IU/day)	76.0 (50.0–92.0)	38.0 (22.0–54.0)	38.0 (22.0–44.0)	0.002*
HbA1c (%) (mmol/mol)	6.8 (6.4–7.5)	6.5 (6.2–6.7)	6.8 (6.6–7.0)	0.102
	51.0 (46.0–58.0)	48.0 (44.0–50.0)	51.0 (49.0–53.0)	
FPG (mmol/L)	5.8 (5.0–6.2)	6.6 (5.0–6.76)	6.6 (6.2–6.6)	0.018*
Fasting C-pep (nmol/L)	0.5 (0.4–0.7)	0.5 (0.4–0.6)	0.4 (0.3–0.7)	0.368
Glucagon stimulated C-pep (nmol/L)	0.9 (0.4–1.3)	0.7 (0.6–0.9)	0.8 (0.5–1.0)	0.867
HOMA-IR (%)	1.2 (0.8–1.7)	1.3 (0.9–1.5)	0.9 (0.7–1.8)	0.565
HOMA-β (%)	69.4 (59.6–107.5)	60.2 (49.2–78.6)	52.3 (41.4–77.5)	0.651
HOMA-S (%)	83.7 (59.0–118.1)	79.8 (69.0–104.1)	107.9 (56.0–147.5)	0.565
Hypoglycemic episodes/patient/month	0.0 (0.0–3.0)	8.0 (5.0–11.0)	0.0 (0.0–3.0)	0.013*
Mixed meal tolerance test				
Fasting C-peptide (nmol/L)	0.5 (0.3–0.7)	–	0.3 (0.2–0.5)	0.612
AUC of C-peptide (nmol/L)	179.9 (77.4–251.5)	–	151.9 (123.4–206.8)	1.000
Peak C-peptide (nmol/L)	1.8 (1.1–3.3)	–	2.2 (1.6–2.6)	1.000
Hyperglycemic clamp study				
Fasting C-pep (nmol/L)	0.6 (0.5–0.8)	–	0.6 (0.4–0.9)	0.605
AUC of 1st-phase C-peptide (nmol/L)	4.1 (1.9–5.9)	–	4.5 (2.9–6.8)	0.553
AUC of 2nd-phase C-peptide (nmol/L)	52.6 (29.1–70.2)	–	74.0 (35.8–90.1)	0.018*
Insulin sensitivity index (µmoles/kg/min/pmol/L)	0.2 (0.1–0.4)		0.2 (0.1–0.2)	0.066
Hyperinsulinemic–euglycemic clamp study				
Glucose disposal rate (mg/kg min)	2.4 (1.9–2.9)	–	2.2 (1.9–2.8)	0.139
Insulin sensitivity (mg/kg min/μU/mL)	3.4 (2.5–6.0)	–	3.4 (2.6–4.6)	0.553

All values are expressed as median and interquartile range

* Significant difference from baseline

The glucagon stimulated C-peptide, AUC and peak C-peptide response to mixed meal tolerance test did not show any significant alteration after ABM-MNCs transplantation. Similarly, no alteration was observed in HOMA-IR, HOMA-β and HOMA-S at the end of the study (Table 1).

The β-cell function as assessed by hyperglycemic clamp study at baseline and AUC of the 1st phase C-peptide response were not significantly different before and after ABM-MNCs transplantation; whereas AUC of the 2nd phase C-peptide response was significantly augmented after ABM-MNCs transplantation as compared to the baseline (p = 0.018). However, the insulin sensitivity index (ISI) did not differ after the procedure (Table 1). Glucose disposal rate and insulin sensitivity as assessed by hyperinsulinemic–euglycemic clamp showed no significant difference from the baseline (Table 1).

### Adverse events

One patient had nausea and vomiting following glucagon administration. None of the patients developed major hypoglycemia, however, median incidence of minor hypoglycemic episodes was eight episodes per patients per month at 3 months, which was significantly reduced at 6 months (Table 1).

## Discussion

This is the first study to examine the alterations in glucose–insulin homeostasis by various dynamic tests including metabolic studies in patients with T2DM after ABM-MNCs transplantation. Our study demonstrates that ABM-MNCs transplantation resulted in reduction of insulin requirement by ≥50%, while maintaining HbA1c < 7.0% (<53.0 mmol/mol). This was substantiated by significant improvement in 2nd phase C-peptide response on hyperglycemic clamp study, while there was no improvement in insulin sensitivity as measured by the hyperinsulinemic–euglycemic clamp. However, no other dynamic test could help in predicting the outcome.

Few studies have demonstrated the utility of ABM-MNCs transplantation in patients with T2DM. Estrada et al. [8] observed that use of autologous bone marrow-derived stem cell infusion into the dorsal pancreatic artery along with hyperbaric oxygen therapy (HBO) resulted in a significant reduction in fasting plasma glucose and insulin doses, and an increase in fasting C-peptide levels at the end 12 months. However, inclusion of subjects with varying degree of glycemic control at baseline, lack of placebo arm and only one-third of the patients completed the study, were the major limitations.

Another study by Wang et al. showed combined use of ABMSCT and HBO in 31 patients with T2DM resulted in decrease in HbA1c of >1.5% as early as 4 weeks following therapy, and it was maintained over the study period of 1 year. The mixed meal tolerance test showed a significant increase in C-peptide response at 3 months but declined to baseline after 1 year of follow-up. It was concluded that the combined.

ABMSCT and HBO therapy resulted in improvement in blood glucose control, accompanied by a reduction in insulin requirement and oral anti-diabetic drugs, and a transient improvement in β-cell function [9].

Our previous study showed ≥50% reduction in the insulin requirement from the baseline in 70% of the patients with a reduction in HbA1c by 1.1% accompanied by a significant increase in glucagon stimulated C-peptide and no significant difference in HOMA-IR after ABMSCT. This reduction in insulin requirement and the change in HbA1c persisted for at least 15 months in approximately two-thirds of the patients. However, in this study, there was no placebo group, and the patients had uncontrolled diabetes with a mean HbA1c of 8.4% at baseline as hyperglycemic milieu per se may influence the stemness of the stem cells [6]. Another study from our center demonstrated a significant reduction in insulin requirement ≥50%, while maintaining HbA1c ≤ 7%, with an improvement in glucagon stimulated C-peptide at 6 and 12 months in patients treated with ABMSCT. There was also a significant reduction in the insulin doses in the control group; however, none of the patients could achieve ≥50% reduction in insulin doses and there was no improvement in fasting and stimulated C-peptide [5].

Previous studies have assessed β-cell function in patients with T2DM after stem cell transplantation by fasting C-peptide, HOMA-β, stimulated C-peptide in response to glucagon and mixed meal tolerance test [5–10]. These studies including ours, have also demonstrated variable improvement in β-cell function [5–7, 10]. This is because that mixed meal tolerance test does not provide precise information on the 1st and the 2nd phase of insulin secretion, because glucose concentration is variable throughout the experiment. So, to normalize insulin secretion to varying glucose level, mathematical model e.g. insulinogenic index are used [16]. Glucagon stimulation test also does not measure any phase of insulin secretion and insulin secretory response is elicited by a non-glucose stimulus [16]. HOMA-β is calculated by a single-time-point measurement of plasma glucose and insulin, and it cannot reflect the complex relationship between variables in glucose-insulin feedback system. Further, these homeostasis model indices progressively loses their importance in a patients with advancing duration of T2DM, as plasma insulin level is the numerators for these measures which progressively declines with increasing duration of diabetes [17]. The hyperglycemic clamp is to clamp the blood glucose at higher target level, which is fundamentally to stimulate insulin secretion based on the feed-back system. This method demonstrates both the 1st and the 2nd phase of insulin secretion. Earlier studies have used various measures to assess β-cell function after stem cell transplantation in vivo, but the data for the clamp studies in human are not available.

We have used various methods to assess the β-cell function and insulin sensitivity. The reduction in insulin requirement was associated with significant improvement in AUC of 2nd phase C-peptide response on a hyperglycemic clamp and no improvement in insulin sensitivity on hyperinsulinemic–euglycemic clamp after the administration of MNCs. However, glucagon stimulation test, mixed meal tolerance test and HOMA-β did not demonstrate improvement in C-peptide response.

The lack of control arm in our study raises the possibility of “placebo-like effect” of ABM-MNCs. However, our previous study [10] could establish that ABM-MNCs had a significant decrease in insulin dose requirement accompanied by an increase in C-peptide response, while the control group had a modest decrease in insulin requirement for a short period of time and was not accompanied by an increase in C-peptide response.

The strengths of our study include the use of the best possible method for the assessment of β-cell function and insulin sensitivity following stem cells transplantation. The limitations include small sample size, short duration of follow-up and a lack of control group.

In conclusion, ABM-MNCs transplantation in patients with T2DM results in significant reduction in insulin requirement accompanied by an improvement in β-cell function as assessed by hyperglycemic clamp. Metabolic clamp studies may be more useful than conventional indices to predict β-cell function or insulin sensitivity in patients with advanced duration of T2DM.

## Additional file

**Additional file 1: Table S1.** Details of stem cell therapy procedure.

## Abbreviations

ABM-MNCs: autologous bone marrow-derived mononuclear cells
T2DM: type 2 diabetes mellitus
HbA1c: glycated hemoglobin
AUC: area under curve
HOMA-IR: homeostatic model assessment of insulin resistance
HOMA-β: homeostatic model assessment of β cell function
HOMA-S: homeostatic model assessment of insulin sensitivity
ABMSCT: autologous bone marrow-derived stem cells transplantation
MSCs: mesenchymal stem cells
MNCs: mononuclear stem cells
