# Supplementary material for: Autologous bone marrow-derived mononuclear cells transplantation in type 2 diabetes mellitus: effect on β-cell function and insulin sensitivity
Source: Diabetol Metab Syndr. 2017 Jul 4;9:50. doi: 10.1186/s13098-017-0248-7 (PMC5496640; doi:10.1186/s13098-017-0248-7)
Supplement: Supplementary file 1 — Additional file 1: Table S1. Details of stem cell therapy procedure. [file 13098_2017_248_MOESM1_ESM.docx]

**Table S1- Details of stem cell therapy procedure**

| **Parameters** | **MNCs**  **(n=7)** |
| --- | --- |
| **Volume of bone marrow aspirated (mL)** | **224.0(213.0-227.0)** |
| **Aspiration** | |
| **Unilateral** | **4** |
| **Bilateral** | **3** |
| **Cell count ( x 10^9^)** | **1.2(1.0-1.4)** |
| **Procedure Time (min)** | **40.0(25.0-47.0)** |
| **Artery injected** | |
| **Superior pancreaticoduodenal** | **7** |

All values are expressed as median and interquartile range.
